# Unsupervised detection of rare events in liquid biopsy assays

**DOI:** 10.1038/s41698-025-01015-3

**Published:** 2025-07-05

**Authors:** Javier Murgoitio-Esandi, Dean Tessone, Amin Naghdloo, Stephanie N. Shishido, Brian Zhang, Haofeng Xu, Agnimitra Dasgupta, Jeremy Mason, Rajiv M. Nagaraju, George Courcoubetis, James Hicks, Peter Kuhn, Assad A. Oberai

**Affiliations:** 1 Department of Aerospace and Mechanical Engineering, Viterbi School of Engineering, University of Southern California, Los Angeles, CA USA; 2 Convergent Science Institute for Cancer, Michelson Center, University of Southern California, Los Angeles, CA USA; 3 Department of Biological Sciences, Dornsife College of Letters, Arts and Sciences, University of Southern California, Los Angeles, CA USA; 4 Department of Computer Science, Viterbi School of Engineering, University of Southern California, Los Angeles, CA USA; 5 Institute of Urology, Catherine & Joseph Aresty Department of Urology, Keck School of Medicine, University of Southern California, Los Angeles, CA USA; 6 Norris Comprehensive Cancer Center, Keck School of Medicine, University of Southern California, Los Angeles, CA USA; 7 Department of Biomedical Engineering, Viterbi School of Engineering, University of Southern California, Los Angeles, CA USA

**Keywords:** Cancer screening, Mathematics and computing

## Abstract

The use of liquid biopsies in the detection, diagnosis and treatment monitoring of different types of cancers and other diseases often requires identifying and enumerating instances of analytes that are rare. Most current techniques that aim to computationally isolate these rare instances or events first learn the signature of the event, and then scan the appropriate biological assay for this signature. While such techniques have proven to be very useful, they are limited because they must first establish what signature to look for, and only then identify events that are consistent with this signature. In contrast to this, in this study, we present an automated approach that does not require the knowledge of the signature of the rare event. It works by breaking the assay into a sequence of components, learning the probability distribution of these components, and then isolating those that are rare. This is done with the help of deep generative algorithms in an unsupervised manner, meaning without a-priori knowledge of the rare event associated with an analyte. In this study, this approach is applied to immunofluorescence microscopy images of peripheral blood, where it is shown that it successfully isolates biologically relevant events in blood from normal donors spiked with cancer-related cells and in blood from patients with late-stage breast cancer.

## Introduction

Liquid biopsy (LBx) has demonstrated the feasibility and clinical utility of blood-based cancer detection through applications in early detection, disease monitoring, and treatment management^1–7^. Studies have shown that even asymptomatic patients can exhibit detectable levels of cancer-associated analytes in the blood^8–12^. These analytes include acellular components such as cell-free DNA, RNA, proteins, extracellular vesicles, and cellular components like circulating cancer cells and tumor microenvironment cells. While cell-based detection approaches have been shown to identify a wide spectrum of cancer-related cells, they may struggle to scale into clinical practice due to the high degree of human involvement required for evaluating each assay in order to identify these rare events.

Circulating tumor cell (CTC) counts have been demonstrated to have prognostic value^1–3^ and predictive utility^4,13–15^, while CTC characterization has shown substantial heterogeneity in both phenotype^16–19^ and genotype^20–23^. Specific biological features, such as protein marker expression, have been found to be critical for therapeutic decision-making^4,7^. However, the field has been limited to either enumeration approaches of CTCs in clinical trials or limited biological characterization in clinical studies. While enumeration approaches have demonstrated clinical utility, biological characterization connects primary tumors to metastatic disease in ways that could offer deeper clinical insights.

Several sample preparation methods have been developed^1,2,24^, each of which produces image datasets of target cells (cancer-related cells) mixed with non-target immune cells, often at ratios as extreme as 1 in 1 million. These imaging results require extensive human interpretation, typically performed by a pathology-trained technician supported by computational algorithms, which require significant prior knowledge about features that are biologically relevant. This restricts the scalability across multiple disease systems and laboratories.

Beyond scalability limitations, the heterogeneity of biomarkers emerging from LBx highlights the need for more generalizable analyte classification and discovery tools. Within the cancer cell population, various phenotypes–including platelet-coated CTCs^7^ (CTCs that have platelets attached), epithelial-to-mesenchymal transition (EMT) CTCs^25^ (cells transitioning from an epithelial to a mesenchymal state), and CTC clusters^26–30^ (aggregates of CTCs)–have emerged as powerful predictive biomarkers in prostate, breast, lung, colorectal, and other cancers. Additionally, increasing evidence has demonstrated the presence of various tumor microenvironment cells in the blood of cancer patients at clinically relevant levels, including circulating endothelial cells^31^ and cancer-associated fibroblasts^32^, which can serve as companion biomarkers to traditional CTCs. Methods that enrich for a specific cellular population limit the ability to detect the heterogeneity of known circulating cancer-associated cells and to discover novel biomarkers in the LBx. Further, if multiple classes of events are deemed important, methods that can detect each class must be developed, which can be a difficult task as it requires large amounts of labeled data. These factors necessitate approaches that can accommodate biomarker diversity without relying on significant prior knowledge. With this as motivation, we present an automated, unsupervised approach that does not require the prior specification or knowledge of a relevant or interesting event. Instead, the approach operates under the principle that these events tend to be rare, and then develops a method for identifying a small cohort of the most rare events without any supervision regarding what these rare events are.

In machine learning, the task of identifying rare events is often referred to as anomaly detection. Unsupervised anomaly detection is carried out without any prior knowledge regarding which events are rare and is accomplished by two broad categories of techniques. The first includes methods that explicitly evaluate the probability density (or log-density) of a given sample. This is done by transforming the sample of interest from its native probability measure to a known, reference measure, and computing the Jacobian of this transformation. The transformation may be achieved by energy-based models (EBMs)^33^, normalizing flows (NFs)^34^, and score-based diffusion models^35^. For an application of these models to anomaly detection the reader is referred to^36,37^. The evaluation of the probability (or log-probability) typically requires computing the Jacobian of the transformation, which makes these techniques computationally expensive.

The second category of anomaly detection methods includes those that train an autoencoder (AE) to reproduce events from the distribution of interest, and then use the reconstruction error as a metric of rarity^38–40^. AEs are a class of unsupervised learning generative models with two components: an encoder and a decoder. The encoder network reduces the dimensionality of the input data to an *n*-dimensional vector (latent vector), and the decoder network reconstructs the input data from the latent vector. The models are trained to maximize the ability to reconstruct the input data with minimal information loss in the latent vector encoding. The logic behind using these for anomaly detection is that the AE learns to reconstruct common events more accurately, as they are the supermajority of the training set, and produces a larger reconstruction error for rare events. When compared with techniques that directly compute the probability, these techniques are computationally efficient but lack the underlying rigorous justification.

**Fig. 1:**
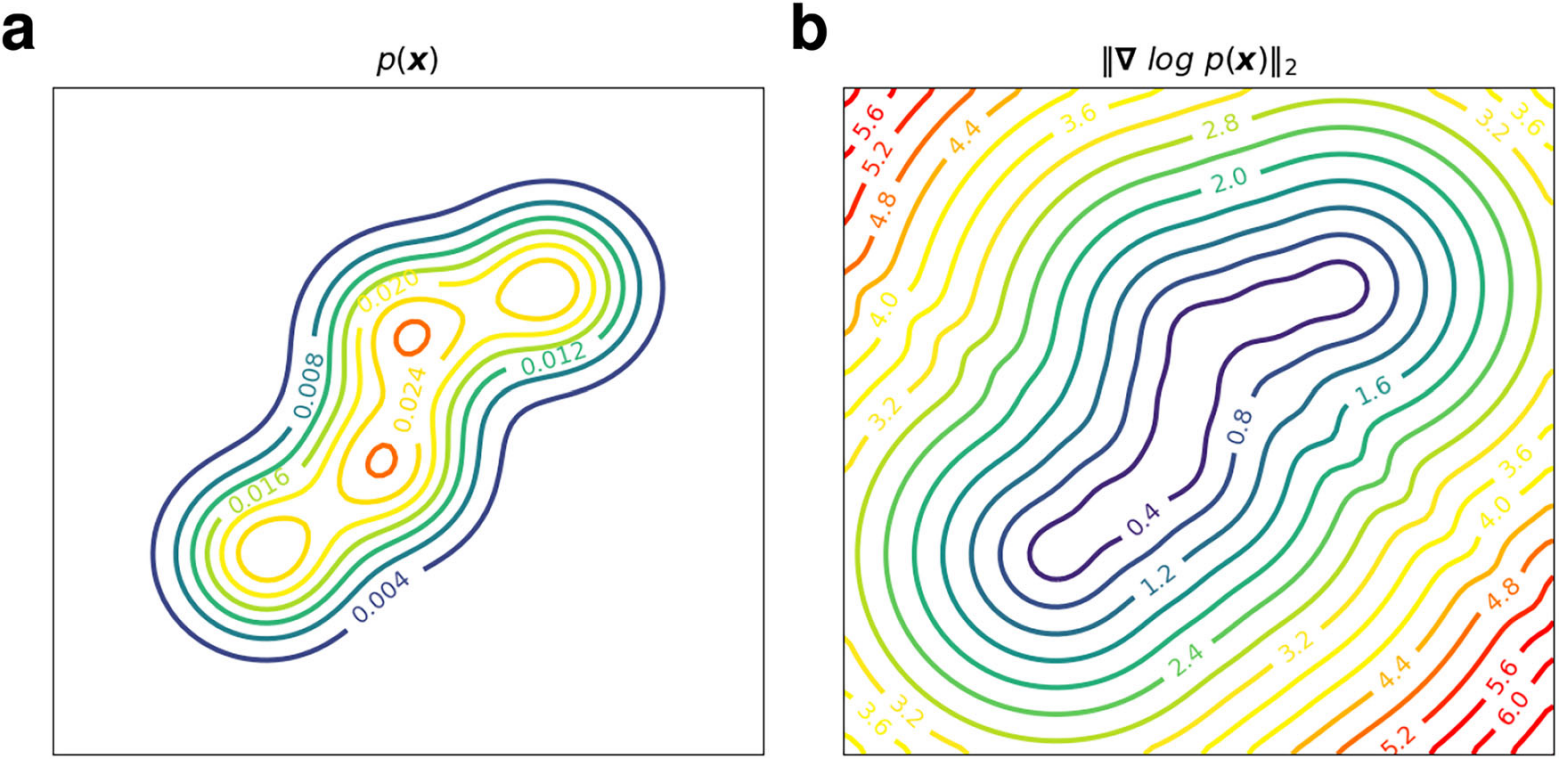
Relation between probability density and its score for a typical Gaussian mixture. **a** Iso-contours of the probability density function (pdf) of a Gaussian mixture model in two dimensions. **b** Iso-contours of the magnitude of the score function for the same pdf. Note that the score function is large in regions where the density is small.

This issue can be addressed by training a special type of AE called the denoising autoencoder (DAE) and using its reconstruction error as a metric for rarity. DAEs are designed to reconstruct the original data from a noisy version of the data; it can be shown that the reconstruction error for a DAE approximates the magnitude of the score function (the gradient of the logarithm, $\nabla \log (p)$) of the probability density function^41^ for the data distribution. For most density functions, the magnitude of the score function is small in regions where the probability mass is concentrated (high-density regions) and large in the low-density regions. The magnitude of the score function, therefore, is a good measure of the rarity of an event (see Fig. 1 for example). Motivated by these arguments, in this study we employ a DAE for detecting rare events.

**Fig. 2:**
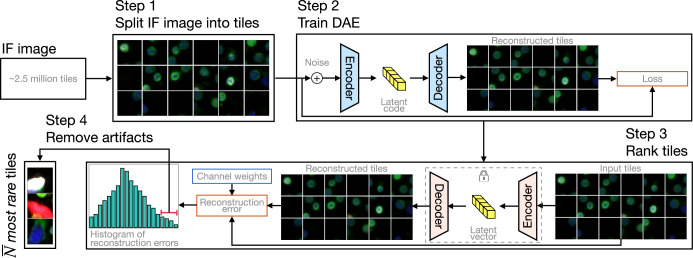
Schematic diagram of the rare event detection (RED) pipeline. In Step 1, one IF image is split into ≈ 2.5 million non-overlapping tiles. In Step 2, pairs of synthetically generated noisy tiles and their clean counterparts are used to train a denoising autoencoder (DAE). In Step 3, noisy tiles are used as input to the trained DAE and the difference between the de-noised and the original clean version of the tiles is used in combination with user-specified IF channel weights to evaluate the reconstruction error for each tile. Tiles with large values of the reconstruction error are identified and are deemed as being rare. In Step 4, an approach that assumes that true rare events are unlikely to be localized to a region within an IF image is used to eliminate artifacts from the rare tile cohort.

Our approach begins by dividing a single four-channel immunofluorescence (IF) image of a slide into approximately *N* ≈ 2.5 million tiles (see Fig. 2). The size of the tile is selected so that each tile contains, on average, up to 4 events, where an event may be a cell, a vesicle or some other blood-based analyte. For applications considered in this study, this yields tiles with 32 × 32 pixels. Thereafter, uncorrelated Gaussian noise is added to each tile and pairs of clean and noisy tiles are used to train a DAE. When the training is complete, each tile is used as input to the DAE and the magnitude of the difference between the output of the DAE and the tile itself is evaluated for each IF channel. This scalar is multiplied with user-supplied channel weights, such that markers with important variance in the assay are emphasized, and the resulting products are summed to yield a single reconstruction error value for each tile. This error is used as a rarity metric to rank the tiles from most rare (largest reconstruction error) to least rare and a cohort $\bar{N}\ll N$ rare tiles is identified. In the final step, an algorithm to remove imaging artifacts from the rare tile cohort is applied and tiles with artifacts are replaced with tiles with slightly lower rarity metric. The approach is described in detail in the Methods section. We refer to this algorithm as the Rare Event Detection algorithm, or the RED algorithm in short.

## Results

In this section we describe the results obtained from applying the RED algorithm to two sets of IF images. The first set corresponds to blood from normal donors that is spiked with two different cell types. This set was used to optimize the RED pipeline configuration by selecting the optimal architecture and hyperparameters (see Section “Denoising autoencoder training”). The second set corresponds to blood from late stage breast cancer patients and was used to evaluate the RED pipeline in a relevant setting. Both sets comprise IF images with four channels representing DAPI (for DNA), a cocktail of cytokeratins (for epithelial cells) labeled with Alexa Fluor 555, vimentin (for mesenchymal cells) labeled with Alexa Fluor 488, and CD45/CD31 (for immune and endothelial cells, respectively) multiplexed in the same channel, labeled with Alexa Fluor 647. In order to keep the notation succinct, we refer to these channels as D, CK, V and CD, respectively. The collection and preparation of the samples, the construction of the assay, and the image acquisition are described in Section 4.1. The subsequent steps that begin with an IF image for a given subject and end with the rank ordering of each tile (defined as a 32 × 32 × 4 sub-region of an image) as per its rarity metric are described in Section 4.2.

In order to assess the utility of the RED algorithm, we adopt the following perspective. We note that a typical IF image contains around *N* ≈ 2.5 million tiles, and most of these contain immune cells that are not biologically interesting. Our hypothesis is that the RED algorithm is able to reduce this number down to a cohort that is about a thousand-fold smaller, $\bar{N}=2500$, without eliminating a significant proportion of biologically interesting cells. The utility of the much smaller rarity-ranked cohort is that it enables manual and automated downstream tasks, including single cell genomics and proteomics, that would not be feasible when working with the original cohort of 2.5 million tiles. Further, it is likely that there is utility in the ranking itself - that is, the fact a tile appears higher in the ranking is likely to be significant - though this remains to be verified in later studies.

For a given value of $\bar{N}$, the rare tile cohort identified by RED represents tiles that have been classified as containing an interesting event. In order to quantify the performance of this classification, we compare this set with an independent set that is determined through an alternate, human-assisted pipeline described in our earlier work^7,25,42^ and summarized in Section 4.2. We refer to this approach as the Outlier Clustering Unsupervised Learning Automated Report (OCULAR) pipeline. In this pipeline, several machine learning algorithms are first used to identify an average of approximately 3000 (range = 1172 to 10,617, mean = 3162, standard deviation = 2676) potentially interesting events in an IF image. This is followed by a step where two human-trained analysts select the biologically interesting events from this reduced set. We treat the set identified by the OCULAR pipeline as the reference ground truth, and report our true and false positive rates (TPR and FPR) relative to this set. Specifically, for both the spiked cell and breast cancer patient examples, the set of tiles identified by OCULAR as interesting is treated as the ground truth positive set. The ground truth negative set is the complement of this set in the slide. Therefore, when evaluating the performance of RED, the set of false positives comprises those tiles that are identified as interesting by RED and belong to the ground truth negative set. The false positive rate is obtained by dividing this number by the total number of ground truth negative tiles. We also vary $\bar{N}$ and construct the receiver operator characteristic (ROC) curve for our approach. We plot the ROC curve and report the area under the curve (AUROC), noting that only the initial part of the curve, where $\bar{N}$ is small, is useful in an application of the RED algorithm.

For the late stage breast cancer patients, we also quantify the performance of the RED algorithm using a human-assisted pipeline. Within this pipeline, the $\bar{N}=2500$ rare tiles identified by the RED algorithm for every subject are examined by two human experts, who extract the biologically interesting events from this cohort. We do this to identify events that were detected by the RED algorithm but not the OCULAR pipeline. We note that although in evaluating the TPR and FPR above OCULAR is treated as a reference ground truth, it is itself not a perfect algorithm. In other words, it has its own FPR, and it is likely that RED may identify novel detections that are outside the set of events identified by OCULAR. In this case, there are two important metrics to assess the performance of the RED algorithm: the fraction of events detected by the OCULAR pipeline that are also detected by the RED algorithm and the number of additional events that are detected by the RED algorithm. We find that the RED algorithm finds 66 out of the 79 events detected by OCULAR; additionally it finds 91 events that are not detected by the OCULAR pipeline. This, along with the fact that it requires minimal manual optimization, clearly illustrates its utility.

### Rare event detection in spiked cell samples

The ND samples with cell lines (SK-BR-3 and HPAEC cell lines) spiked in comprise nine IF slides. Of these nine slides, three are spiked with only SK-BR-3 cells, three are spiked with only HPAEC cells, and three are spiked with both. The SK-BR-3 cells are a model system for rare epithelial cells or CTCs, while the HPAEC cells are a model system for rare endothelial cells. On average, each IF slide contains 342 (min. = 19, max. = 1030) spiked-in cells as identified by the OCULAR pipeline.

**Fig. 3:**
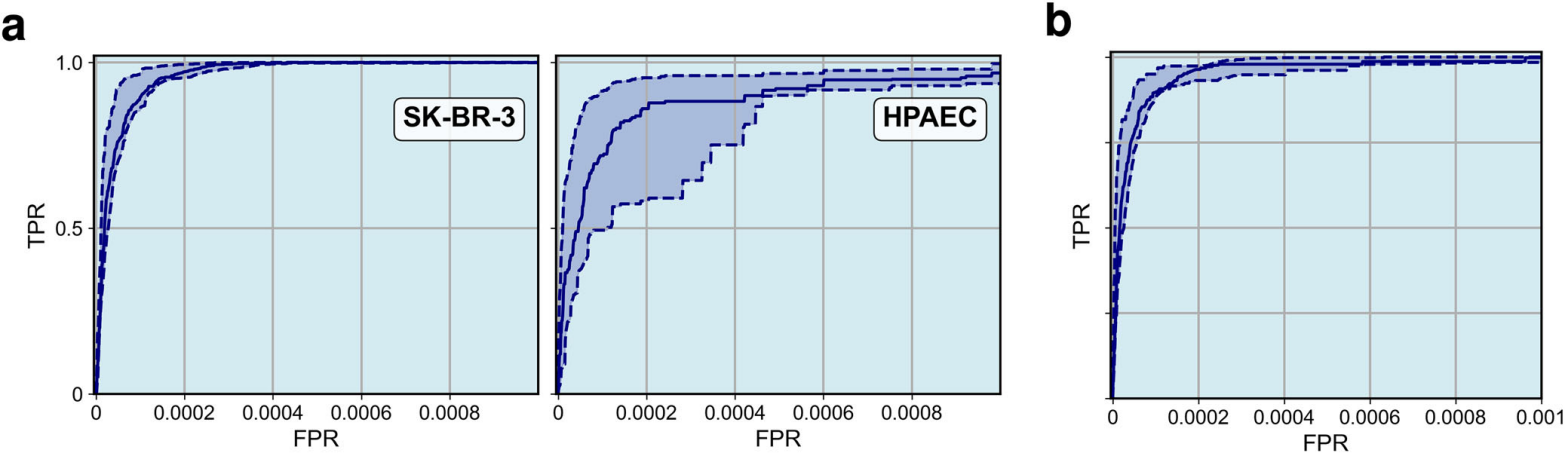
Initial part of the ROC (FPR range from 0 to 0.001) curve for the rare event detection algorithm applied to the spiked cell slides. Subfigure (**a**) shows the ROC curve for SK-BR-3 and HPAEC cell lines separately, while subfigure (**b**) shows the ROC curve for both cell lines. The solid curves represent the median ROC across all subjects, and the dashed curves represent the lower and upper quartiles.

For each IF image we apply the RED algorithm, vary $\bar{N}$ from zero to *N* and compute the ROC curves consisting of the FPR and TPR values for each $\bar{N}$. We do this for each spiked cell type separately and also for both cell types combined. This results in six ROC curves for SK-BR-3, six ROC curves for HPAEC, and nine ROC curves both cell types combined. For each set (SK-BR-3, HPAEC cells, and combined) we evaluate the lower quartile, median, and upper quartile ROC curve values. In Fig. 3 we plot the initial part of these curves (until FPR = 0.001). The solid curve represents the median, and the dashed lines represent the lower and upper quartiles. We observe that in every case the new algorithm yields a mean TPR close to unity (0.993, 0.965, and 0.985) for a very small FPR =0.001. We do not plot the entire ROC curve since the values of the area under the ROC curve (AUROC), which is reported in Table 1, are very close to 1.

**Table 1:** Application of the RED algorithm to the spiked cell dataset

	TPR ($\bar{N}=2,500$)				AUROC			
Cell type	Mean	St. dev.	Min.	Max.	Mean	St. dev.	Min.	Max.
SK-BR-3	0.993	0.014	0.962	1.00	1.00	0.0	1.00	1.00
HPAEC	0.965	0.031	0.927	1.00	0.999	2.00 × 10^−3^	0.993	1.00
All	0.985	0.020	0.943	1.00	0.999	1.00 × 10^−3^	0.997	1.00

Columns 2-5: statistics for the true positive rate for a cohort of 2500 rare tiles identified by the algorithm. Columns 6-9: statistics for the AUROC obtained by varying $\bar{N}$ from 0 to *N*. Values are reported for CTCs (Row 1), endothelial cells (Row 2) and their combination (Row 3).

In Table 1, we report the statistics for TPR across the nine subjects for $\bar{N}=2500$ noting that this value of $\bar{N}$ corresponds to a 1000-fold reduction in data. For both cell types, the value of TPR with this 1000-fold reduction in data is high (mean = 0.993 for SK-BR-3 and mean = 0.965 for HPAEC). Overall, the RED algorithm misses around 1.5% of biologically relevant events even with 1000-fold data reduction. In this table we also report the area under the ROC curve (AUROC) for the two cell types and all spiked cells taken together. The AUROC values obtained are very close to unity. We also observe that for both cell types the performance of the algorithm across slides is consistent (St. dev. for TPR = 0.020) though the variability is higher for HPAEC cells (St. dev. for TPR = 0.031).

**Fig. 4:**
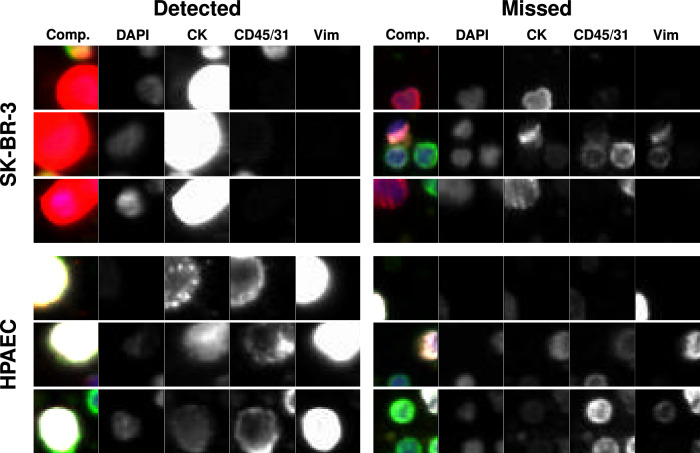
Representative gallery of rare events in samples from normal donors spiked with SK-BR-3 and HPAEC cell lines. For each rare event the composite image is shown followed by the biomarker fluorescent channels (specified by the headers). The top three rows show SK-BR-3 events and the bottom three rows show HPAEC events. The left column shows the events detected by RED and the right column rows shows the events not detected by RED.

In Fig. 4 we plot some of the tiles from the two spiked cell lines (SK-BR-3 and HPAEC) that were detected by the RED algorithm within a cohort of $\bar{N}=2500$ tiles. We also plot some that were missed. We observe that the tiles that were detected tended to contain large, bright pieces of relevant cells, whereas those that were missed contained smaller pieces.

### Detection of rare cells in breast cancer patients

The late-stage breast cancer set comprises eleven IF labeled slides with each slide representing a sample from a unique late-stage breast cancer patient. On average each IF slide contains 8 (min. = 2, max. = 14) biologically relevant events as identified by the OCULAR pipeline. These biologically relevant events can be grouped into seven categories based on signal in the following channels: D-∣CK, D∣CK, D∣CK∣V, D∣CK∣V∣CD, D∣V, D∣V∣CD, and D∣CK∣CD, where any channel label indicates a positive signal in that channel, and D- denotes a DAPI negative signal, indicating acellularity.

**Fig. 5:**
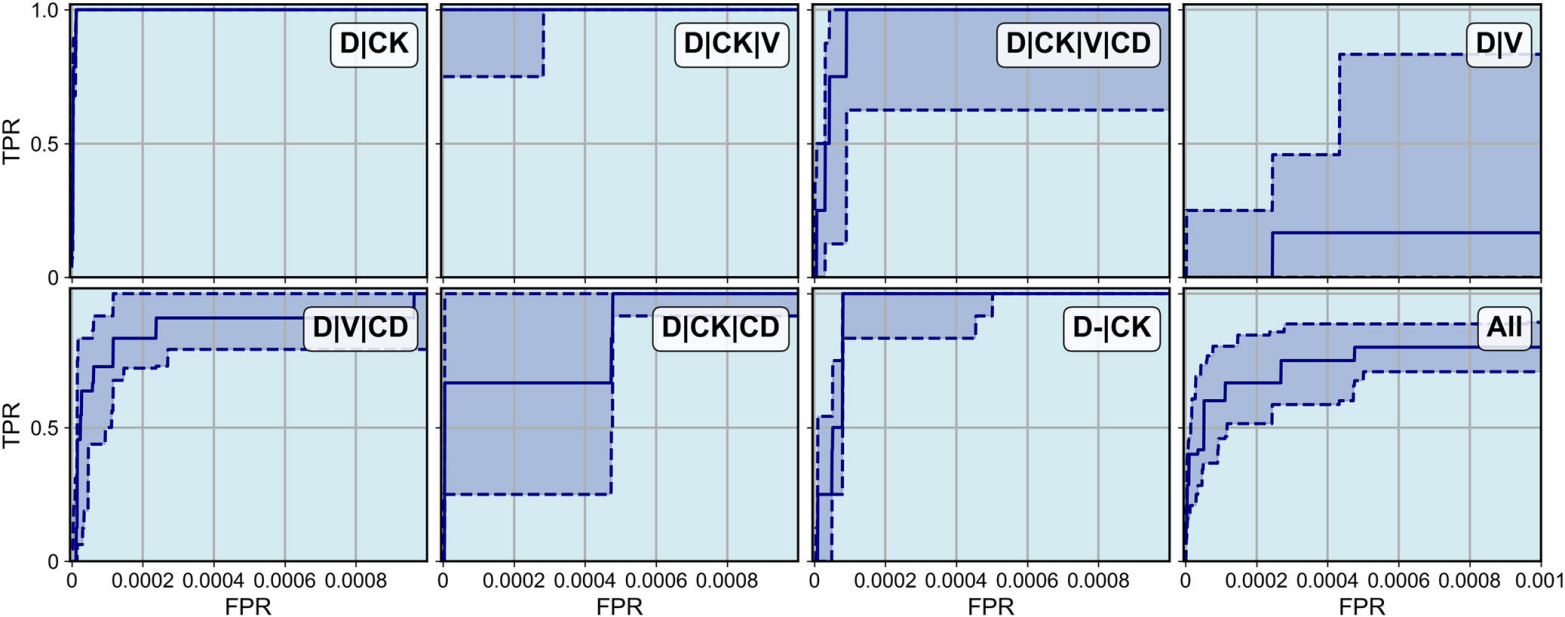
Initial part of the ROC curves for the rare event detection algorithm applied to late stage breast cancer subjects. Separate ROC curves are shown for each event type as well as the composite ROC curve for all event types combined (bottom right). The solid curves represent the median ROC across all subjects, and the dashed curves represent the lower and upper quartiles.

**Fig. 6:**
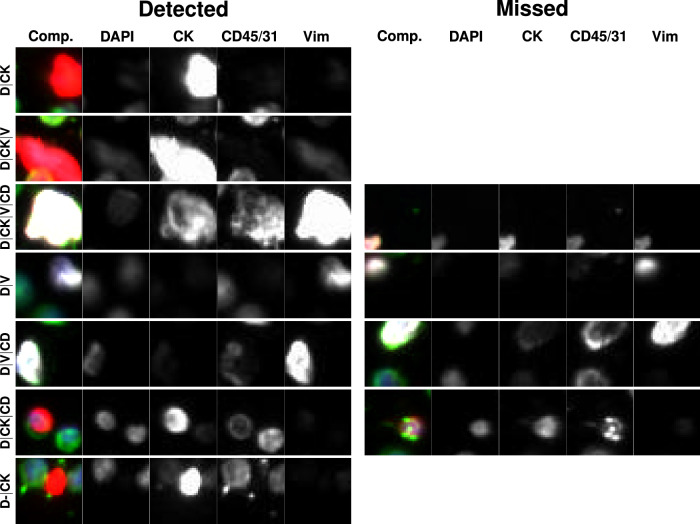
Representative gallery of rare events in samples collected from patients diagnosed with late-stage breast cancer. For each rare event the composite image is shown followed by the biomarker fluorescent channels (specified by the headers). The left column shows rare events detected by RED and the right column shows rare events not detected by RED. No event is shown for the cell types for which no event was missed.

We apply the RED algorithm to these images, and in Fig. 5, plot the lower quartile, median and upper quartile of the earlier part of the ROC curves (until FPR = 0.001) obtained by varying $\bar{N}$ across all subjects and for the seven event categories, as well as all categories combined. The solid curve represents the median ROC curve while the dashed curves represent the upper and lower quartile variations about the median. We observe that the performance of the RED algorithm for this set is not as good as for the spiked cell set (AUROC = 0.982 across all cell types). Further, there is variability in the performance across different event categories. We observe that the algorithm performs well for some event categories (e.g., D∣CK, D∣CK∣V, and D-∣CK positive events) and is challenged in detecting others (e.g., D∣V positive events). To further illustrate this, Fig. 6 shows a sample of the tiles from the late-stage BC slides that were detected by the RED algorithm within a cohort of $\bar{N}=2500$ tiles and some tiles that were not detected within that cohort. In three out of the seven categories there were no tiles that were missed.

In Table 2, we have reported the TPR for the RED algorithm with a thousand fold reduction in data ($\bar{N}=2500$). We observe that for a thousandfold data reduction, the median TPR across all event categories is 0.746, which is lower than the corresponding value for the spiked cell set. This can be attributed to the uncertainty in defining what constitutes a biologically relevant event in cases where these events occur naturally (as in the late-stage breast cancer set) and are not introduced artificially (as in the spiked cell set). This makes the detection of these events difficult for the RED algorithm as well as OCULAR pipeline, which is the approach used as the reference. We also observe that in this case performance across different slides is more variable with a St. dev. in TPR = 0.265. In addition to inherent uncertainty, this may be attributed to the small number of ground truth positive events for some slides. This includes one slide with a single positive event. This event was not captured by the RED algorithm in the top 2500 events yielding TPR = 0 for this slide.

**Table 2:** Application of the rare event detection algorithm to images from late-stage breast cancer subjects

	TPR ($\bar{N}=2500$)				AUROC			
Cell type	Mean	St. dev.	Min.	Max.	Mean	St. dev.	Min.	Max.
D∣CK	1.00	0.0	1.00	1.00	1.00	0.0	1.00	1.00
D∣CK∣V	1.00	0.0	1.00	1.00	1.00	1.00 × 10^−4^	1.00	1.00
D∣CK∣V∣CD	0.750	0.382	0.0	1.00	0.914	0.185	0.500	1.00
D∣V	0.389	0.448	0.0	1.00	0.978	0.0427	0.883	1.00
D∣V∣CD	0.798	0.339	0.0	1.00	0.991	0.0210	0.940	1.00
D∣CK∣CD	0.917	0.144	0.667	1.00	0.998	3.20 × 10^−3^	0.993	1.00
D-∣CK	1.00	0.0	1.00	1.00	1.00	1.00 × 10^−4^	1.00	1.00
All	0.746	0.265	0.0	1.00	0.982	0.0280	0.915	1.00

Columns 2-5: statistics for the true positive rate for a cohort of 2500 rare tiles identified by the algorithm. Columns 6-9: statistics for the AUROC obtained by varying $\bar{N}$ from 0 to *N*. Values are reported for different event types (Rows 1-7) and all types together (Row 8).

A manual examination of the set of 2500 events identified by the RED algorithm revealed that this set included several events that were biologically relevant but were not identified by the OCULAR pipeline. In hindsight, we should have anticipated this since the OCULAR pipeline also has its own false negative errors. This led us to consider the approach described below for quantifying the performance of the RED algorithm.

As described in Section 4.2, the OCULAR pipeline consists of two distinct stages. In the first stage, all events in a given IF slide are segmented and a short-list comprising approximately 3000 interesting events is identified by the OCULAR algorithm. Events in this short-list are then examined by multiple human experts and those deemed to be biologically interesting by both experts are included in the final list of biologically relevant events. Analogous to this, we develop and implement the RED pipeline where the 2500 events per IF slide identified by the RED algorithm were examined by two human experts, and those deemed to be biologically interesting by both experts are included in the final list of biologically relevant events.

**Fig. 7:**
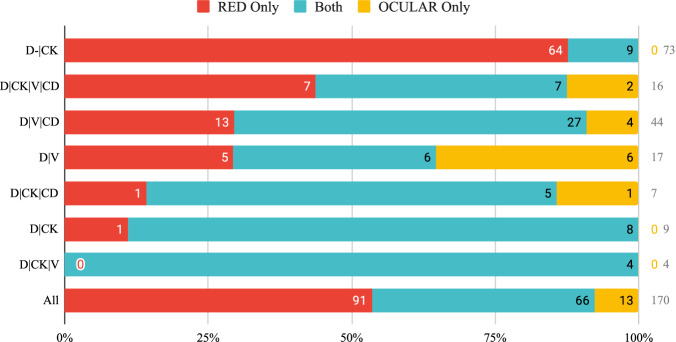
Enumeration of biologically relevant events identified by the RED pipeline alone (in red), the OCULAR pipeline alone (in yellow) and both pipelines (in blue). Rows 1-7 depict results for 7 different event types, while row 8 depicts composite results for all event types.

Once the OCULAR and RED pipelines have identified the set of biologically relevant events, we compute the number of events detected by both pipelines and each pipeline alone. These numbers are reported in Fig. 7. We observe that the RED pipeline identifies around twice as many events when compared with the OCULAR pipeline (157 versus 79). Another way to measure the efficacy of the two pipelines is to consider the number of events identified by only one pipeline. In this respect the RED pipeline identifies seven times as many events as the OCULAR pipeline (91 versus 13). We note that the performance of the RED pipeline is dependent on the event category. In particular, for the D-∣CK category the RED pipeline identifies around 8 times as many events as the OCULAR pipeline (73 vs. 9), while for D∣V events the OCULAR pipeline performs slightly better (11 vs. 12).

## Discussion

The RED algorithm represents a paradigm shift in detecting biologically relevant events in LBx. Most current methods seek specific analytes in LBx assays through physical enrichment. This can be challenging when there is not a single analyte of interest but rather a heterogeneous population. Further, in exploratory studies where the analyte of interest is not known, it is impossible to use these types of methods. In contrast, the RED algorithm works on the simple premise that biologically relevant information is rare relative to the common immune population. This obviates the need to specify the characteristics of what constitutes a biologically relevant event and makes the detection task simpler and easier to automate.

When compared with the baseline approach (OCULAR algorithm), the RED algorithm comprises fewer steps that are easier to automate and require minimal expert guidance. In particular, in the RED algorithm, the steps required to get to the cohort of 2500 rare events are: training the DAE, using the DAE to rank tiles, and removing artifacts through an automated approach. The expert input required for these steps is limited to specifying the channel weights (four scalar values), and the threshold (a single scalar value) used in removing tiles that contain artifacts. In contrast, the steps in OCULAR include threshold segmentation of around 2.5 million events, evaluation of 761 parameters for each event, reduction of these to 350 PCA components, and cascading clustering stages which result in a wide range of events retained for final analysis. These steps require the specification of: (a) hyperparameters for segmentation, (b) each of the 761 features to be computed for each event, (c) number of PCA components to be retained, (d) majority cluster elimination from the cascading cluster stages to help with negative depletion of majority class and (e) distances that constitute an adequately rare event compared to median references. Overall, this requires significantly more information to be specified by a computational expert, which makes this approach harder to automate. Further, the RED algorithm retains only 2500 rare events per IF slide, whereas the OCULAR algorithm retains roughly 3000 events per IF slide, both of which require some level of human data curation. As such, the RED algorithm leads to more significant data reduction, which makes downstream analysis easier and more efficient.

In the spiked cell cohort considered in this study, most of the epithelial and endothelial cells were captured in the set of 2500 rare tiles identified by the RED algorithm. On average it missed 0.7% of the epithelial cells and 3.5% of the endothelial cells. This served to validate the performance of RED in a case where the biologically relevant events were well known and could be easily characterized.

The late stage breast cancer cohort comprised naturally occurring biologically relevant events that were not contrived. In this case, the rare tiles identified by the RED algorithm were examined by two experts in order to select biologically interesting events. The performance of this pipeline, which was dubbed the RED pipeline, was compared with that of a similar analysis which used OCULAR to identify the rare events. It was found that the RED pipeline yielded twice as many biologically relevant events, which points to its utility in real-world applications. It was also found that the RED algorithm was able to detect most of the events detected by the OCULAR pipeline (84%, Fig. 7).

Additionally, it is clear that the performance of the RED pipeline relative to the OCULAR pipeline varies depending on the event category in question. For the samples considered in this study, RED performed significantly better than OCULAR for D∣CK events, and slightly worse than for D∣V events. This may be attributed to the fact that CK positive events, whether they are biologically relevant or not, are rare in the peripheral blood context, and can therefore be easily identified by a rarity detection algorithm like RED. In contrast to this, V-positive events are common, with a population of leukocytes expressing vimentin, and a very small fraction of these cells (V-positive with variable expression in the other channels) are biologically relevant. In this case a rarity detection algorithm has to work “harder” relying on factors like cell morphology and relative intensity across multiple channels in order to detect biologically relevant rare events. Overall, the analysis shows that the RED pipeline performs better than the baseline method OCULAR for every event category except D∣V where its performance is marginally worse (11 versus 12 events identified).

The RED methodology offers improved sensitivity over the baseline OCULAR method. RED identifies a greater number of rare events, which is critical for enhancing detection capabilities in a rarity-focused framework. This methodology is particularly advantageous because it is largely automated, reducing human involvement and thereby minimizing potential sources of error and the time required for analysis. However, it is important to note the limitation of RED, and similar rarity based approaches, in inferring cell type. Molecular characterization of the detected events is essential to elucidate their biological and clinical relevance. For instance, D∣CK cells are consistent with canonical epithelial CTCs, and D∣V∣CD cells are morphologically and phenotypically consistent with circulating endothelial cells. Highly multiplexed proteomics, through techniques such as Imaging Mass Cytometry^43^, or single-cell genomics^44^, would be necessary to confirm each cell’s association with a true biological type. Additionally, the D-∣CK events identified by RED are hypothesized to be oncosomes or large extracellular vesicles potentially associated with the disease state^45^. Differentiating these from similarly shaped fluorescent secondary antibody aggregates^46^ is challenging with four-plex fluorescence microscopy alone. Similar downstream proteomics work is necessary to fully characterize this population. Further validation studies will confirm these biological phenotypes and provide deeper insights into their role in cancer biology and potential clinical implications. We note that since the expert annotation is performed only on a filtered set of images (those identified by RED and OCULAR) all evaluation metrics reported reflect performance on this filtered set and do not provide a complete measure of the algorithms’ ability to detect all rare events present in the original, unfiltered datasets. Indeed, the direct approach for computing evaluation metrics over the original unfiltered set would require expert annotation over a very large number of images and is in inherently challenging task these types of studies.

When compared to the CellSearch platform, which is a widely used enrichment-based approach clinically utilized in breast cancer patient care, RED demonstrates distinct advantages. CellSearch is tailored to detect known cell types, specifically circulating tumor cells that are EpCAM+, CK+, and CD45-, and relies on a predefined set of markers. While effective for certain applications, this targeted approach introduces bias and limits the detection of rare and unconventional events, such as oncosomes or tumor microenvironment components like endothelial cells or fibroblasts. In contrast, RED’s unbiased framework allows for the identification of a broader range of rare events, enabling novel discoveries and expanding the potential applications of liquid biopsy. These attributes position RED as a transformative tool in rare event detection, with the capacity to uncover previously undetected facets of disease biology.

Single-channel biophysical enrichment approaches, while streamlined, often result in the loss of multidimensional enrichment capabilities, which are crucial for capturing the complex heterogeneity of rare events. This limitation underscores the importance of a methodology like RED, which preserves sensitivity to rare populations without compromising the breadth of detection. This is borne out in the current study where RED is shown to be more sensitive than OCULAR, the framework used by the HDSCA platform, which has in turn demonstrated more sensitivity than CellSearch in detecting cellular heterogeneity and plasticity^47–49^. Moreover, RED offers a distinct advantage from a development perspective. Its algorithmic design simplifies the process of enriching rare event populations, making it “lightweight” and user-friendly for developers. This reduced dependency on deep biological understanding allows researchers to focus on refining detection and analysis pipelines rather than grappling with complex enrichment processes.

## Methods

### Blood collection, sample preparation and imaging

Peripheral blood (PB) samples were collected in cell-free DNA blood collection tubes (Streck, La Vista, NE USA) and processed as previously described^47,48,50^. Briefly, after complete blood cell count (Medonic M-series Hematology Analyzer, Clinical Diagnostic Solutions Inc., Fort Lauderdale FL USA) the red blood cells were lysed with ammonium chloride and all nucleated cells were plated as a monolayer on custom cell adhesion glass slides (Marienfeld, Lauda, Germany) at approximately 3 million cells per slide, followed by blocking with 7% bovine serum albumin (BSA) before drying and cryopreservation at -80 ^∘^C.

All cancer patients were enrolled between April 2013 and January 17, 2017, at multiple clinical sites in the United States: Billings Clinic (Billings, MT), Duke University Cancer Institute (Durham, NC), City of Hope Comprehensive Cancer Center (Duarte, CA), and University of Southern California Norris Comprehensive Cancer Center (Los Angeles, CA). Research involving human research participants, material, or data were performed in accordance with the Declaration of Helsinki. Patient recruitment took place according to an institutional review board-approved protocol at each site and all study participants provided written informed consent. This study was approved by the University of Southern California, University Park Institutional Review Board (FWA 00007099, USC UPIRB #UP-14-00523).

Samples were stained automatically (IntelliPATH FLX autostainer, Biocare Medical LLC) with the Landscape immunofluorescence (IF) assay as previously published^5,9,25,42,51–53^. Briefly, slides were thawed and fixed with 2% paraformaldehyde prior to 1) incubation with anti-human CD31 Alexa Fluor 647 direct conjugate (mouse IgG1 monoclonal antibody; 2.5 *μ*g/mL; clone: WM59; Cat# MCA1738A647; BioRad; RRID:AB 322463) and anti-mouse Fab fragments (IgG goat monoclonal; 100 *μ*g/mL; Cat# 115-007-003; Jackson ImmunoResearch), 2) permeabilization with cold methanol, 3) incubation with a mixture of anti-human pan cytokeratin (CK) (CKs 1,4,5,6,8,10,13,18,19 mouse IgG1/IgG2a monoclonal antibody cocktail; 210 *μ*g/mL; Cat# C2562; clone: C-11, PCK-26, CY-90, KS-1A3, M20, A53-B/A2; Sigma; RRID:AB 476839), anti-human CK 19 (mouse IgG1 monoclonal antibody; 0.2 *μ*g/mL; Cat# GA61561-2; clone: RCK108; Dako), anti-human CD45 Alexa Fluor 647 direct conjugate (mouse IgG2a monoclonal antibody; 1.2 *μ*g/mL; Cat# MCA87A647; clone: F10-89-4; AbD Serotec; RRID:AB 324730), and anti-human vimentin (VIM) Alexa Fluor 488 direct conjugate (rabbit IgG monoclonal antibody; 3.5 *μ*g/mL; Cat# 9854 BC; clone: D21H; Cell Signaling Technology; RRID:AB 10829352), and 4) incubated with anti-mouse IgG1 Alexa Fluor 555 (goat IgG polyclonal antibody; 2 *μ*g/mL; Cat# A21127; Invitrogen; RRID:AB 141596) and 4’,6-diamidino-2-phenylindole (DAPI; dilution: 1: 50,000; Cat# D1306; Thermo Fisher Scientific; RRID:AB 2629482). Slides were mounted with a glycerol-based media, coverslipped, and sealed.

Automated scanning was done at 100X magnification using a custom high-throughput fluorescence scanning microscope across 2304 frames per slide in each channel (DAPI, Alexa Fluor 488, Alexa Fluor 555, Alexa Fluor 647). The exposure time and gain per channel were automatically set to ensure consistent background intensity across all slides for normalization purposes.

Normal donor (ND) samples were procured from the Scripps Normal Blood Donor Service and processed according to the above. Cell line cells with known expression profiles were spiked into the sample at various concentrations after red blood cell lysis (SK-BR-3 ATCC HTB-30 and HPAEC ATCC PCS-100-022). Standard protocols were followed for contrived sample analysis.

A total of 11 samples collected from patients with metastatic breast cancer were included in this study. Patient recruitment took place according to an institutional review board-approved protocol approved by the University of Southern California (FWA 00007099, USC UPIRB #UP-14-00523) and all study participants provided written informed consent^47,54^.

### Rare event detection (RED) algorithm

In order to detect rare events within the IF assay, we employ a deep learning method for anomaly detection. In the first step of our approach we split the IF image for a subject into a set of non-overlapping sub-images that we refer to as tiles (see Fig. 2). The size of a tile is selected so that each tile includes 1–4 cells on average. In our case, this corresponds to a size 32 by 32 pixels, or 18.9 by 18.9 *μ*m, which yields approximately 2.5 million tiles per IF image.

The collection of tiles generated is used to train a denoising autoencoder (DAE). During training, the input to the DAE is a noisy version of each tile and its output is the corresponding de-noised version. The noisy version of a tile is generated by artificially adding uncorrelated homoscedastic Gaussian noise (with variance = 0.05^2^) to every pixel of the tile. Note that the noise is added to tiles with pixel values normalized between 0 and 1. The DAE learns how to reconstruct tiles that contain common events well, but not tiles that contain rare events. Consequently, when tiles with common events are passed through the fully trained DAE it produces images that are close to the original tile, whereas for tiles with rare events this is not the case. The magnitude of the difference between the reconstructed tile and the original tile is computed on a per-channel basis. This magnitude is then multiplied with a channel-dependent weight and all the weighted values are added to arrive at a single real-valued reconstruction error, which is used as the rarity metric. The values of weights used in this study are 1/3 for the DAPI, CK and V channels, and 0 for the CD channel. Note that the DAE does not use any labeled data during training the DAE or when computing the rarity metric for each tile. Thus, our approach is unsupervised and works without any a-priori information regarding biologically relevant events, such as location in the IF assays or phenotype, specific to the disease.

To ensure rarity scoring emphasizes true rare events rather than the dominant white blood cell population, we set the CD reconstruction weight to zero in our evaluation of tile rarity. CD45/CD31 is highly expressed on leukocytes, which make up the vast majority of circulating cells. The predominant populations studied as outliers in liquid biopsies are canonical epithelial circulating tumor cells (CTCs), mesenchymal CTCs, additional derivatives of CTCs (such as clusters), and circulating CAFs. These cells are defined by expression of cytokeratins, vimentin, and DAPI channels, such that weighting those channels ensures improved recovery of tumor-associated phenotypes in rarity scoring. We determined the optimal channel weights experimentally by spiking SK-BR-3 tumor cells and HPAEC endothelial cells into normal blood, testing all channel weight combinations, and selecting the configuration that maximized recovery of these contrived rare cells while minimizing false outlier calls among white blood cells.

Tiles with large values of the reconstruction error are deemed as rare, where those with small values are deemed as being common. There is a theoretical justification of this observation. It can be shown that for a given input sample, the reconstruction error is an approximation of the magnitude of the score function of the underlying probability density for that sample^41^. Further, since for most probability densities the magnitude of the score function is much larger in regions where the probability mass is small, the reconstruction error may be used as a metric for rarity.

During the application of the proposed approach we observed that some tiles that contain imaging artifacts were selected in the rare tile cohort. This is not surprising given the understanding that certain types of imaging artifacts can also be rare. In the examples considered in this manuscript the artifacts include speck-like regions with a strong signal in CK channel, and blurs and streaks across all channels. Both these artifacts tended to occur in clusters at a specific location of the image, and this characteristic was used to remove the tiles with these artifacts. For the specklike artifacts, the number of artifacts occurring within a sub-domain of an image was counted, and if this number exceeded a specified threshold, all tiles in the rare event cohort from that subdomain were removed. This subdomain was set to 1362 by 1004 pixels, the original size of the images taken by the scanning microscope, and the threshold used was 500 specks per subdomain. To eliminate other regionally concentrated artifacts present in the rare tile cohort, the number of tiles from the top 10,000 rare tiles per subdomain was calculated. If this number exceeded 25, all tiles from that subdomain were removed. This approach is based on the observation that artifacts tend to be regionally concentrated, whereas biologically significant events are dispersed throughout the IF image. Hence, removing a few subdomains (typically less than 2% of the image) has negligible effect on the biological signal while effectively removing artifacts from the top of the ranking. Note that the subdomains used for artifact removal are predefined and are non-overlapping.

### Denoising autoencoder training

The hyperparameters for the denoising autoencoder (DAE) were selected by optimizing its performance in detecting rare events in spiked cell samples (see Section 2.1). This optimization targeted three key hyperparameters: the variance of the added noise (*σ*^2^), the number of training epochs (*N*_epochs_), and the dimension of the latent space (*z*). The optimal values identified were *σ* = 0.05, *N*_epochs_ = 1, and *z* = 512. The value *N*_epochs_ = 1 may appear to be small when compared with typical values used in deep learning; however, the reader should keep in mind that our training set is very large (around 2.5 million samples), hence, a single epoch is sufficient for the DAE to learn the features of a “typical” tile. For each slide, the DAE is trained for one epoch using the Adam optimizer with a learning rate of 10^−5^ and a batch size of 500.

The autoencoder model consists of an encoder and a decoder, with each composed of convolutional, dense, pooling, and upsampling layers. The details of the encoder and decoder architectures are described in Table 3.

**Table 3:** Autoencoder architecture: encoder and decoder layers

Encoder	Decoder
Conv2D(4, 32) - ReLU	Linear(512, 512) - ReLU
Dense-block(32, 3)	Linear(512, 1536) - ReLU - BN
Conv2D(32, 64) - AvgPool2D(2, 2) - ReLU	Linear(1536, 2048) - ReLU
Dense-block(64, 3)	Reshape(2, 2, 512)
Conv2D(64, 128) - AvgPool2D(2, 2) - ReLU	Conv2D(512, 256) - ReLU - Upsample(2, 2)
Dense-block(128, 3)	Dense-block(256, 3)
Conv2D(128, 256) - AvgPool2D(2, 2) - ReLU	Conv2D(256, 128) - ReLU - Upsample(2, 2)
Dense-block(256, 3)	Dense-block(128, 3)
Conv2D(256, 512) - AvgPool2D(2, 2) - ReLU	Conv2D(128, 64) - ReLU - Upsample(2, 2)
Flatten	Dense-block(64, 3)
Linear(2048, 1536) - ReLU - BN	Conv2D(64, 32) - ReLU - Upsample(2, 2)
Linear(1536, 512) - ReLU	Dense-block(32, 3)
Linear(512, 512)	Conv2D(32, 4) - Sigmoid

The layers in the architecture shown in Table 3 are described as follows. Linear(*in, out*) represents a fully connected layer with *in* input dimensions and *out* output dimensions. Conv2D(*in, out*) are 2D convolutional layers with a kernel size of 3, where *in* is the number of input filters and *out* is the number of output filters. AveragePool2D(*pool_size, stride*) is a downsampling layer with the specified pooling size and stride. Dense-block(*k, n*) refers to the block architecture in^55^, with *k* input filters and *n* layers. Upsample(*size, size*) represents an upsampling layer with a scaling factor of *size*. *BN* denotes batch normalization.

The denoising autoencoder was trained using the mean squared error (MSE) loss function in Eq. S (1), as described in ref. ^56^

1 $${\mathcal{L}}(r)=\frac{1}{N}\mathop{\sum }\limits_{i=1}^{N}\parallel {{\boldsymbol{x}}}_{i}-r({{\boldsymbol{x}}}_{i}+{\boldsymbol{\epsilon }}){\parallel }_{2}^{2}.$$

In this equation, ${\{{{\boldsymbol{x}}}_{i}\}}_{i = 1}^{N}$ represents the set of input tiles, and *r*(***x***_*i*_ + ***ϵ***) denotes the autoencoder’s reconstruction of each input tile after adding noise. The noise, ***ϵ***, is sampled independently for each tile from a Gaussian distribution ${\mathcal{N}}({\mathbf{0}},{\sigma }^{2}{\boldsymbol{I}})$, where ***I*** is the identity matrix and *σ*^2^ is the variance of the added noise and is determined through hyperparameter optimization. The DAE was trained on a NVIDIA V100 GPU, with each slide taking approximately 30 minutes to train.

**Fig. 8:**
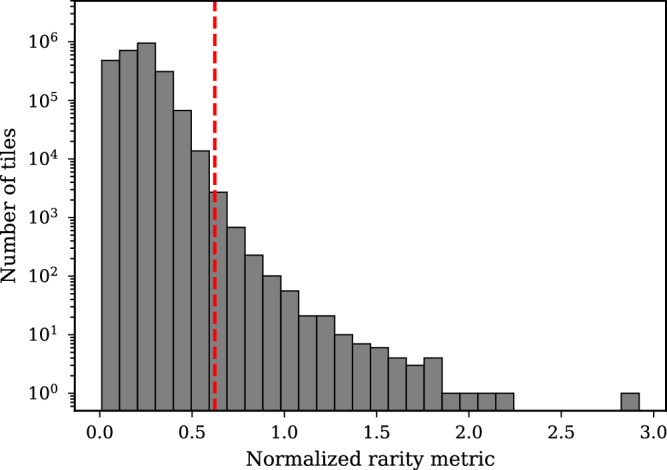
Histogram of the rarity metric across tiles from a late-stage breast cancer sample, where the rarity metric is normalized by the average magnitude of the tiles for the subject. The red dashed line indicates the threshold for rare tile detection that yields 2500 rare tiles for this subject.

Figure 8 presents the histogram of the normalized rarity metric for tiles from a representative late-stage breast cancer subject obtained by normalizing the rarity metric by the average value of the norm of intensity of input tiles. In order to make the plot readable, we have plotted the frequency (number of tiles) using a log scale. The vertical dashed line at a value of 0.624 represents the threshold used to define a rare tile. The 2500 tiles that are included in the bins to the right of this threshold are considered rare. The average rarity metric for the rare tiles is 0.732 while for the complementary set it is 0.206.

### OCULAR rare event algorithm

In this study OCULAR was used as a reference to quantify the performance of the RED algorithm. OCULAR is a custom algorithm for rare event detection used in the high-definition single cell assay (HDSCA) workflow that uses image processing for feature extraction, dimensionality reduction, and unsupervised clustering^7,25,42^. Namely, the “EBImage” package (EBImage 4.12.2) is used to segment the fluorescent images for every event across the slide, separating cells (expressing DAPI) from acellular components (not expressing DAPI). This is followed by feature extraction for each cell, generating 761 quantitative parameters across the 4 IF channels and paired combinations of each. A principal component analysis (PCA) transform is calculated and each cell’s morphometric data is projected onto the top 350 principal components. This reduction was shown to retain 99.95% of the original variance. Next, event-to-event distances for all cells in a given frame are calculated and ≈ 30 hierarchical clusters are generated. Thereafter, a cell is defined as rare if it belongs to the smallest clusters until the number of cells added by including a cluster exceeds 1.5% of all events on a frame or if it is far away from the median event in a frame. After this frame-based identification, frames are clustered into 10 bins based on their aggregated feature values. Events in each group are first compared internally, where rare events are filtered based on distance to common event clusters, and then further filtered with the same method when aggregated with the whole slide. Around 3000 cellular events are initially identified as rare and potentially interesting.

In the OCULAR pipeline, the events identified by the OCULAR algorithm described above are examined by two experts and those deemed as being biologically relevant by both experts are retained.

### Comparison of RED and OCULAR pipelines

To compare the algorithms, we sought to identify biologically interesting events found through each method. First, two human experts evaluated composite RGB images and single-channel grayscale images for the OCULAR results, determining whether the events were biologically relevant. Events that both experts agreed were relevant were retained for a total of 113 OCULAR events. Next, the 2500 rarest tiles as identified by RED were examined by one human expert as both composite RGB images and single-channel grayscale images to determine an initial subset of 609 potentially interesting tiles. The main criterion in selecting an upper bound of 2500 tiles as interesting was determined from the results presented in Fig. 5 where we have plotted the ROC curve for each cell type and for all types taken together. The largest value of FPR = 0.001 included in this plot corresponds to a set of 2500 tiles. As can be seen from these plots, the TPR reaches it maximum value and ceases to change after an FPR of about 0.0005, which corresponds to around 1250 tiles. Recognizing this and allowing for the fact that another 1250 tiles beyond this number may also contain interesting tiles, we selected an upper bound of 2500. Moreover, the 2500 selected rare tiles represent a 1000-fold reduction from the original data containing approximately 2.5 million tiles per slide. A further 29 tiles that corresponded to OCULAR events that were missed in this evaluation were added to the potentially interesting tiles. Both experts independently evaluated each tile for biological relevance and tiles rated as irrelevant by either or both experts were removed. Tiles that captured components of the same event were also deduplicated, leaving 166 events. Both experts then categorized all events, where disagreements were resolved by deference to one expert or selection of the majority class in cases of events found in both pipelines. Finally, events categorized as D and D∣CD, which are often biologically semi-interesting, as well as one event categorized as D-∣CK∣V∣CD, were removed from both pipelines, leaving 157 RED tiles and 79 OCULAR events. Figure 7 illustrates the events identified by both algorithms, as well as the overlap in identified events, separated by channel classification.

We evaluated the reliability of expert classifications using Cohen’s kappa, a measure for interrater reliability that accounts for chance agreement. This metric ranges from 0 to 1, with 0 indicating no agreement and 1 indicating perfect agreement. Based on all classified events, including D and D∣CD events, we found *κ* = 0.775 ± 0.058 (95% CI), or moderate agreement^57^. This level of agreement illustrates the level of difficulty even for expert human curators to identify cell phenotypes and events of interest.

## Data Availability

A representative spiked cell sample image is available at 10.6084/m9.figshare.29110163.408. Other data will be provided upon request.

## Code availability

The code to train the DAE and rank tiles according to the rarity metric is licensed under the MIT License and is available at https://github.com/jmurgoitioesandi/Unsupervised_RareCellDetection/tree/main/DAE_RED_TF2. Standard Python and matplotlib methods were used to generate the visualizations.

## References

[1] Cristofanilli, M. et al. Circulating tumor cells, disease progression, and survival in metastatic breast cancer. *N. Engl. J. Med.* **351**, 781–791 (2004). 15317891 10.1056/NEJMoa040766

[2] Allard, W. J. et al. Tumor cells circulate in the peripheral blood of all major carcinomas but not in healthy subjects or patients with nonmalignant diseases. *Clin. Cancer Res.* **10**, 6897–6904 (2004). 15501967 10.1158/1078-0432.CCR-04-0378

[3] Howard, D. et al. A sample preparation and analysis system for identification of circulating tumor cells. *J. Clin. Ligand Assay.* **25**, 104–10 (2002).

[4] Scher, H. I. et al. Assessment of the validity of nuclear-localized androgen receptor splice variant 7 in circulating tumor cells as a predictive biomarker for castration-resistant prostate cancer. *JAMA Oncol.* **4**, 1179–1186 (2018). 29955787 10.1001/jamaoncol.2018.1621 PMC6139066

[5] Setayesh, S. M. et al. Multianalyte liquid biopsy to aid the diagnostic workup of breast cancer. *NPJ Breast Cancer* **8**, 112 (2022). 36167819 10.1038/s41523-022-00480-4 PMC9515081

[6] Shishido, S. N. et al. Liquid biopsy landscape in patients with primary upper tract urothelial carcinoma. *Cancers* **14**, 3007 (2022). 35740671 10.3390/cancers14123007 PMC9221424

[7] Chai, S. et al. Platelet-coated circulating tumor cells are a predictive biomarker in patients with metastatic castrate-resistant prostate cancer. *Mol. Cancer Res.* **19**, 2036–2045 (2021). 34462330 10.1158/1541-7786.MCR-21-0383 PMC12747416

[8] Anagnostou, V. & Velculescu, V. E. Pushing the boundaries of liquid biopsies for early precision intervention. *Cancer Discov.* **14**, 615–619 (2024). 38571422 10.1158/2159-8290.CD-24-0037

[9] Resnick, K. et al. Circulation of rare events in the liquid biopsy for early detection of lung mass lesions. *Thoracic Cancer* **15**, 2100–2109 (2024). 39233479 10.1111/1759-7714.15429 PMC11471425

[10] Goebel, C. et al. Diagnosis of non-small cell lung cancer for early stage asymptomatic patients. *Cancer Genomics Proteom.* **16**, 229–244 (2019). 10.21873/cgp.20128 PMC6609262 31243104

[11] Bettegowda, C. et al. Detection of circulating tumor dna in early-and late-stage human malignancies. *Sci. Transl. Med.* **6**, 224ra24–224ra24 (2014). 24553385 10.1126/scitranslmed.3007094 PMC4017867

[12] Crosby, D. Delivering on the promise of early detection with liquid biopsies. *Br. J. Cancer* **126**, 313–315 (2022). 35013576 10.1038/s41416-021-01646-w PMC8811021

[13] Scher, H. I. et al. Association of ar-v7 on circulating tumor cells as a treatment-specific biomarker with outcomes and survival in castration-resistant prostate cancer. *JAMA Oncol.* **2**, 1441–1449 (2016). 27262168 10.1001/jamaoncol.2016.1828 PMC5206761

[14] Scher, H. I. et al. Nuclear-specific ar-v7 protein localization is necessary to guide treatment selection in metastatic castration-resistant prostate cancer. *Eur. Urol.* **71**, 874–882 (2017). 27979426 10.1016/j.eururo.2016.11.024 PMC5401782

[15] Scher, H. I. et al. Validation of nuclear-localized AR-V7 on circulating tumor cells (CTC) as a treatment-selection biomarker for managing metastatic castration-resistant prostate cancer (mCRPC). *J Clin Oncol* **36**, 273–273 (2018).

[16] Scher, H. I. et al. Phenotypic heterogeneity of circulating tumor cells informs clinical decisions between ar signaling inhibitors and taxanes in metastatic prostate cancer. *Cancer Res.* **77**, 5687–5698 (2017). 28819021 10.1158/0008-5472.CAN-17-1353 PMC5666339

[17] Capuozzo, M., Ferrara, F., Santorsola, M., Zovi, A. & Ottaiano, A. Circulating tumor cells as predictive and prognostic biomarkers in solid tumors. *Cells* **12**, 2590 (2023). 37998325 10.3390/cells12222590 PMC10670669

[18] Sinkala, E. et al. Profiling protein expression in circulating tumour cells using microfluidic western blotting. *Nat. Commun.* **8**, 14622 (2017). 28332571 10.1038/ncomms14622 PMC5376644

[19] Reza, K. K. et al. In situ single cell proteomics reveals circulating tumor cell heterogeneity during treatment. *ACS nano* **15**, 11231–11243 (2021). 34225455 10.1021/acsnano.0c10008

[20] Yates, L. R. et al. Genomic evolution of breast cancer metastasis and relapse. *Cancer Cell* **32**, 169–184 (2017). 28810143 10.1016/j.ccell.2017.07.005 PMC5559645

[21] Welter, L. et al. Treatment response and tumor evolution: lessons from an extended series of multianalyte liquid biopsies in a metastatic breast cancer patient. *Mol. Case Stud.* **6**, a005819 (2020). 10.1101/mcs.a005819 PMC7784493 33203646

[22] Gasch, C. et al. Heterogeneity of epidermal growth factor receptor status and mutations of kras/pik3ca in circulating tumor cells of patients with colorectal cancer. *Clin. Chem.* **59**, 252–260 (2013). 23136247 10.1373/clinchem.2012.188557

[23] Owen, S. et al. Simultaneous single cell gene expression and egfr mutation analysis of circulating tumor cells reveals distinct phenotypes in nsclc. *Adv. Biosyst.* **4**, 2000110 (2020). 10.1002/adbi.202000110 PMC7883301 32700450

[24] Moore, H. C. et al. A randomized trial of fulvestrant, everolimus, and anastrozole for the front-line treatment of patients with advanced hormone receptor–positive breast cancer, swog s1222. *Clin. Cancer Res.* **28**, 611–617 (2022). 34844978 10.1158/1078-0432.CCR-21-3131 PMC9782801

[25] Chai, S. et al. Identification of epithelial and mesenchymal circulating tumor cells in clonal lineage of an aggressive prostate cancer case. *NPJ Precis. Oncol.* **6**, 41 (2022). 35729213 10.1038/s41698-022-00289-1 PMC9213535

[26] Carlsson, A. et al. Circulating tumor microemboli diagnostics for patients with non–small-cell lung cancer. *J. Thorac. Oncol.* **9**, 1111–1119 (2014). 25157764 10.1097/JTO.0000000000000235 PMC4145608

[27] Larsson, A.-M. et al. Longitudinal enumeration and cluster evaluation of circulating tumor cells improve prognostication for patients with newly diagnosed metastatic breast cancer in a prospective observational trial. *Breast Cancer Res.* **20**, 1–14 (2018). 29884204 10.1186/s13058-018-0976-0 PMC5994056

[28] Schuster, E. et al. Better together: circulating tumor cell clustering in metastatic cancer. *Trends Cancer* **7**, 1020–1032 (2021). 34481763 10.1016/j.trecan.2021.07.001 PMC8541931

[29] Reduzzi, C. et al. Circulating tumor cell clusters are frequently detected in women with early-stage breast cancer. *Cancers* **13**, 2356 (2021). 34068368 10.3390/cancers13102356 PMC8153325

[30] Yang, Y. et al. Circulating tumour cell clusters: isolation, biological significance and therapeutic implications. *BMJ Oncol.* **3**, 1–14 (2024). 10.1136/bmjonc-2024-000437 PMC11557725 39886139

[31] Welter, L. et al. Cell state and cell type: Deconvoluting circulating tumor cell populations in liquid biopsies by multi-omics. *Cancers* **15**, 3949 (2023). 37568766 10.3390/cancers15153949 PMC10417732

[32] McCarthy, J. B., El-Ashry, D. & Turley, E. A. Hyaluronan, cancer-associated fibroblasts and the tumor microenvironment in malignant progression. *Front. Cell Dev. Biol.* **6**, 48 (2018). 29868579 10.3389/fcell.2018.00048 PMC5951929

[33] LeCun, Y. et al. A tutorial on energy-based learning. *Predicting Structured Data* **1**, 191–241 (2006).

[34] Kingma, D. P. & Dhariwal, P. Glow: Generative flow with invertible 1x1 convolutions. *Adv. Neural Inform. Process. Syst.* **31,** (2018).

[35] Song, Y. et al. Score-based generative modeling through stochastic differential equations. In *International Conference on Learning Representations* (2021).

[36] Zhai, S., Cheng, Y., Lu, W. & Zhang, Z. Deep structured energy based models for anomaly detection. In *International conference on machine learning*, 1100–1109 (PMLR, 2016).

[37] Nachman, B. & Shih, D. Anomaly detection with density estimation. *Phys. Rev. D.* **101**, 075042 (2020).

[38] Yu, J., Zheng, X. & Liu, J. Stacked convolutional sparse denoising auto-encoder for identification of defect patterns in semiconductor wafer map. *Comput. Ind.* **109**, 121–133 (2019).

[39] An, J. & Cho, S. Variational autoencoder based anomaly detection using reconstruction probability. *Spec. Lect. IE* **2**, 1–18 (2015).

[40] Zhang, X. et al. Unsupervised surface anomaly detection with diffusion probabilistic model. In *Proc. of the IEEE/CVF International Conference on Computer Vision*, 6782–6791 (2023).

[41] Vincent, P. A connection between score matching and denoising autoencoders. *Neural Comput.* **23**, 1661–1674 (2011). 21492012 10.1162/NECO_a_00142

[42] Shishido, S. N. et al. Characterization of cellular and acellular analytes from pre-cystectomy liquid biopsies in patients newly diagnosed with primary bladder cancer. *Cancers* **14**, 758 (2022). 35159025 10.3390/cancers14030758 PMC8833768

[43] Giesen, C. et al. Highly multiplexed imaging of tumor tissues with subcellular resolution by mass cytometry. *Nat. Methods* **11**, 417–422 (2014). 24584193 10.1038/nmeth.2869

[44] Baslan, T. et al. Genome-wide copy number analysis of single cells. *Nat. Protoc.* **7**, 1024–1041 (2012). 22555242 10.1038/nprot.2012.039 PMC5069701

[45] Gerdtsson, A. S. et al. Large extracellular vesicle characterization and association with circulating tumor cells in metastatic castrate resistant prostate cancer. *Cancers* **13**, 1056 (2021). 33801459 10.3390/cancers13051056 PMC7958848

[46] Donaldson, J. G. Unit 4.3 immunofluorescence staining. *Current protocols in cell biology/editorial board, Juan S. Bonifacino...[et al.]* Unit (2001). 10.1002/0471143030.cb0403s00 PMC4709840 18228363

[47] Shishido, S. N. et al. Preanalytical variables for the genomic assessment of the cellular and acellular fractions of the liquid biopsy in a cohort of breast cancer patients. *J. Mol. Diagnostics* **22**, 319–337 (2020). 10.1016/j.jmoldx.2019.11.006 PMC7103765 31978562

[48] Seo, J. et al. Plasticity of circulating tumor cells in small cell lung cancer. *Sci. Rep.* **13**, 11775 (2023). 37479829 10.1038/s41598-023-38881-5 PMC10362013

[49] Narayan, S. et al. Defining a liquid biopsy profile of circulating tumor cells and oncosomes in metastatic colorectal cancer for clinical utility. C*ancers (Basel)* **14**, 4891 (2022). 10.3390/cancers14194891 PMC9563925 36230811

[50] Marrinucci, D. et al. Fluid biopsy in patients with metastatic prostate, pancreatic and breast cancers. *Phys. Biol.* **9**, 016003 (2012). 22306768 10.1088/1478-3975/9/1/016003 PMC3387996

[51] Shishido, S. N. et al. Determining the efficacy of exthera seraph100 blood filtration in patients diagnosed with pancreatic cancer through the liquid biopsy. *BJC Rep.* **2**, 47 (2024). 39516545 10.1038/s44276-024-00069-3 PMC11524105

[52] Ghoreifi, A. et al. Blood-based liquid biopsy: A promising noninvasive test in diagnosis, surveillance, and prognosis of patients with upper tract urothelial carcinoma. In *Urologic Oncology: Seminars and Original Investigations*, vol. 42, 118–e9 (Elsevier, 2024). 10.1016/j.urolonc.2024.02.001 38383240

[53] Shishido, S. N. et al. Cancer-related cells and oncosomes in the liquid biopsy of pancreatic cancer patients undergoing surgery. *npj Precis. Oncol.* **8**, 36 (2024). 38360856 10.1038/s41698-024-00521-0 PMC10869814

[54] Rodríguez-Lee, M. et al. Effect of blood collection tube type and time to processing on the enumeration and high-content characterization of circulating tumor cells using the high-definition single-cell assay. *Arch. Pathol. Lab. Med.* **142**, 198–207 (2018). 29144792 10.5858/arpa.2016-0483-OA PMC7679174

[55] Huang, G., Liu, Z., Van Der Maaten, L. & Weinberger, K. Q. Densely connected convolutional networks. In *Proc.of the IEEE conference on computer vision and pattern recognition*, 4700–4708 (2017).

[56] Alain, G. & Bengio, Y. What regularized auto-encoders learn from the data-generating distribution. *J. Mach. Learn. Res.* **15**, 3563–3593 (2014).

[57] McHugh, M. L. Interrater reliability: the kappa statistic. *Biochemia Med.* **22**, 276–282 (2012). PMC3900052 23092060

